# Retroperitoneal cavernous lymphangioma in an adult: a case report and review of the literature

**DOI:** 10.3389/fonc.2026.1832278

**Published:** 2026-04-22

**Authors:** Sailing Fu, Chengshi Deng, Guangjia Liu, Zhixi Lin, Tingde Long, Wenxue Wu, Baofa Luo

**Affiliations:** 1 Department of Medical Imaging, People’s Hospital of Wenshan Prefecture, Wenshan, Yunnan, China; 2 Department of Pathology, People’s Hospital of Wenshan Prefecture, Wenshan, Yunnan, China

**Keywords:** adult, case report, cavernous lymphangioma, imaging findings, retroperitoneal

## Abstract

Lymphangiomas are rare benign lesions that seldom occur in the abdominal cavity. Retroperitoneal involvement is particularly uncommon and accounts for only 1% of all cases. We reviewed cases of retroperitoneal lymphangiomas reported over the past decade, among which cystic lymphangiomas were the most prevalent subtype. This study reports the clinicopathological characteristics and imaging findings of a case of primary retroperitoneal cavernous lymphangioma. Due to its atypical imaging manifestations, the preoperative diagnosis was retroperitoneal sarcoma. Through this case report, we aim to provide clinicians with additional insights into the pathological and imaging features of this disease.

## Introduction

Lymphangiomas are rare benign lesions originating from the lymphatic system. They occur predominantly in children and infants, with relatively fewer cases in adults. These lesions are most commonly located in the neck or axilla. Approximately 5% occur in the abdominal cavity, and only about 1% arise in the retroperitoneum (1). Lymphangiomas are associated with congenital factors such as abnormal development or obstruction of lymphatic vessels. They may also arise from acquired factors, including trauma, inflammation, and other factors (2). The clinical manifestations of retroperitoneal lymphangioma largely depend on the tumor size and the compression exerted on adjacent organs. Patients may manifest symptoms such as abdominal pain, bloating, or intestinal obstruction. In some cases, the condition may remain asymptomatic (3). In rare circumstances, the tumor is discovered following complications such as hemorrhage, infection, or rupture. Computed tomography (CT) and magnetic resonance imaging (MRI) are the most commonly used examination methods for preoperative diagnosis of lymphangioma. These imaging techniques typically reveal unilocular or multilocular cystic masses containing lymphatic fluid with certain specificity. The differential diagnosis includes cystic tumors originating from the ovary, kidney, and pancreas (4), cystic teratomas (5), neurogenic tumors (6), and retroperitoneal hemangiomas (7). When the tumor contains minimal cystic components, imaging alone is not sufficient to provide an accurate diagnosis. We report a case of primary retroperitoneal cavernous lymphangioma with atypical imaging features, which make the preoperative diagnosis challenging. We also review the clinicopathological characteristics and imaging features of lymphangioma, aiming to update the literature on this rare disease and help clinicians improve diagnostic accuracy and treatment strategies while enhancing understanding of this disease.

## Case description

**Figure 1:**
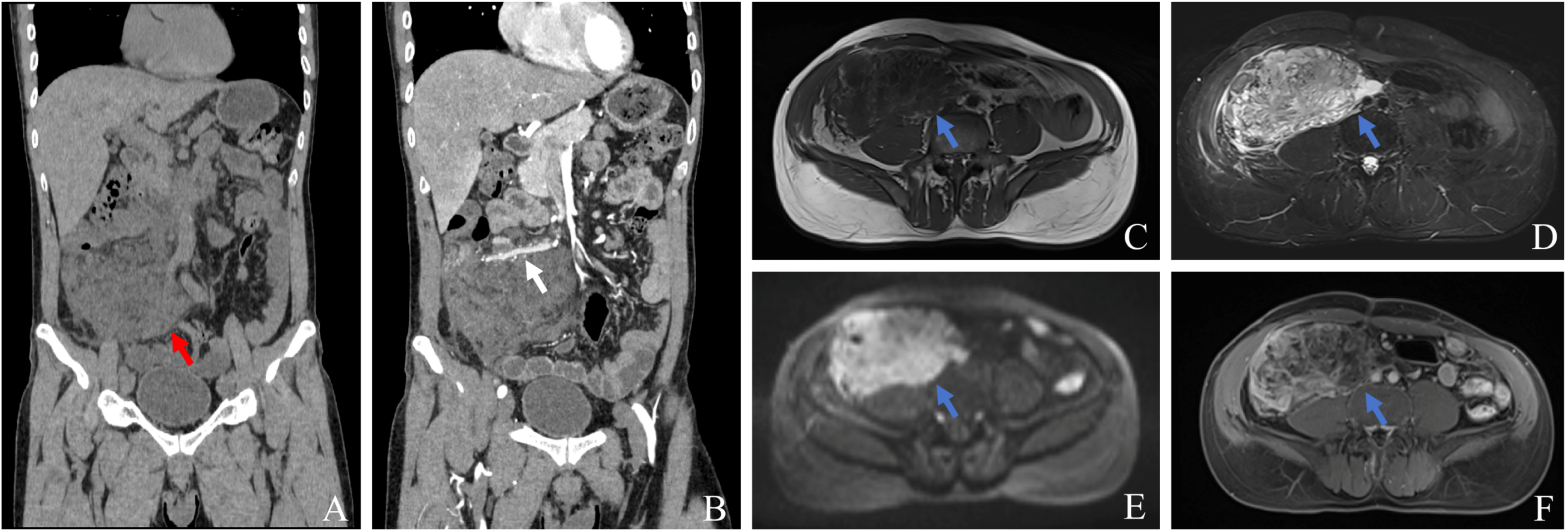
Abdominal CT **(A)** revealed a mixed-density mass (13 × 10.5 × 7.2 cm) in the right lower abdomen with indistinct borders and unclear demarcation from the ileocolonic (red arrow). Contrast-enhanced scan **(B)** illustrates slight thickening of the ileocolic vein with aneurysmal dilatation distally (white arrow). MRI revealed an ill-defined mass with slightly long T1 **(C)**, long T2 **(D)** mixed signal intensity, and high signal intensity on DWI **(E)**, the enhanced scan revealed the lesion enhancement **(F)** (blue arrow).

A 47-year-old male patient was admitted with persistent right lower abdominal pain for more than 3 months without an obvious cause. It was intermittent and accompanied by an abdominal mass. The pain worsened the day before admission. There was no previous history of diabetes mellitus and coronary heart disease. The patient underwent a right lower abdominal tumor resection and a back tumor resection 30 years ago. However, treatment details were unclear due to the long interval. Physical examination revealed tenderness in the right abdomen. A palpable mass measuring approximately 10 × 12 cm was detected. Laboratory tests, including tumor markers (AFP, CEA, CA125, CA153, CA199), were within normal limits. A CT scan revealed a patchy mixed density shadow (13 × 10.5 × 7.2 cm) in the right lower abdomen with unclear boundaries, partially encasing the ileocecal bowel (**Figure 1A**). Enhanced scan revealed slight dilation of the ileocolic vein (**Figure 1B**). MRI demonstrated a mixed-signal mass with slightly longer T1 and T2 signals, and DWI revealed a high signal. The lesion exhibited an unclear border, and solid components demonstrated marked heterogeneous enhancement after contrast administration (**Figures 1C–F**). After exclusion of contraindications to surgery, an exploratory laparotomy was performed. During the operation, a grayish-white mass measuring approximately 13 × 9 × 7.5 cm was observed in the mesentery, with tumor invasion of the anterior abdominal wall, cecum, terminal ileum, and right mesocolon. Under direct visualization, the tumor that invaded the anterior peritoneum was completely dissected using an electric knife, and Toldt’s space was entered. The ascending colon and terminal ileum were transected, followed by a side-to-side ileo-ascending colon anastomosis.

**Figure 2:**
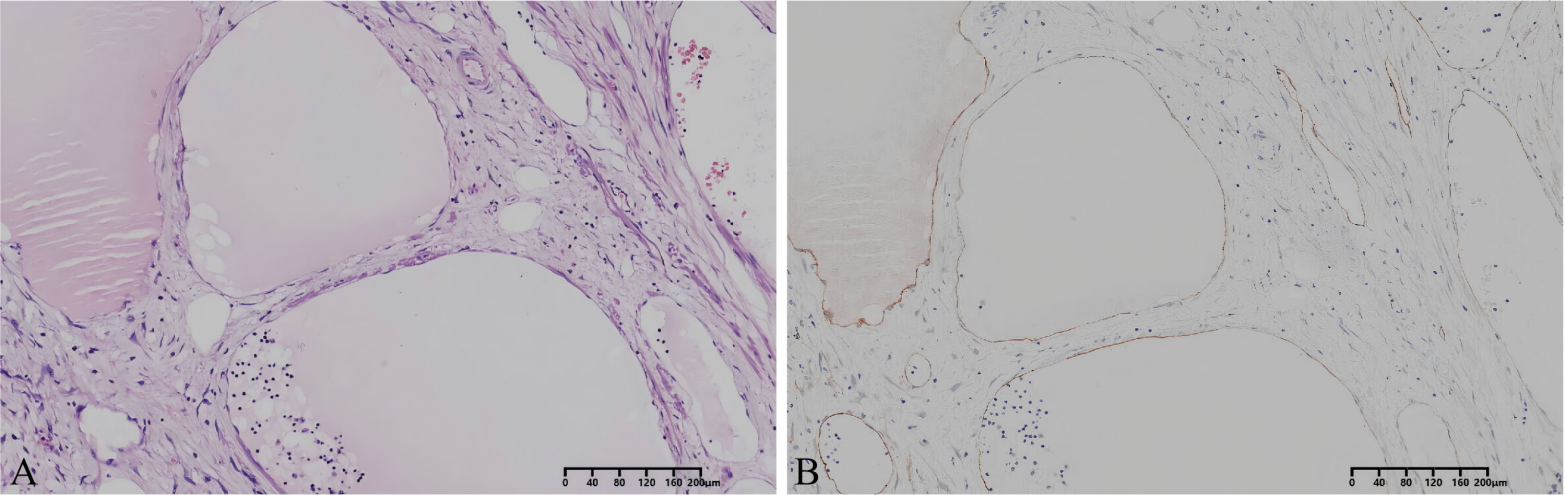
Histopathological findings (×100). Hematoxylin-eosin staining revealed hyperplasia of interstitial fibrous tissue with focal lymphocytic infiltrates **(A)**, immunochemical staining demonstrates positivity for D2-40 **(B)**.

Postoperative pathology revealed a tumor composed of variably sized cystic spaces lined by endothelial cells and filled with lymphatic fluid. These spaces were surrounded by lymphocyte infiltration (**Figure 2A**). Immunohistochemical analysis revealed that the tumor cells were positive for vimentin, CD34, smooth muscle actin (SMA), desmin (partially), Ki-67, and D2-40 (**Figure 2B**). In addition, the tumor cells were negative for PCK, CDK4, MDM2, β-catenin, and S-100 Based on the pathological and immunohistochemical findings, the final diagnosis was retroperitoneal cavernous lymphangioma. The patient recovered well postoperatively. Follow-up imaging evaluation after 6 months revealed no indications of tumor recurrence. Long-term follow-up will be continued.

## Discussion

The pathogenesis of retroperitoneal lymphangioma remains unclear. It is currently believed that this condition results from the isolation of lymphatic tissue during embryonic development. Consequently, the isolated lymphatic tissue fails to establish normal connections with the lymphatic system. This failure leads to progressive accumulation of lymphatic fluid and abnormal dilation of the lumen. Furthermore, impaired lymphatic drainage resulting from trauma, inflammation, and radiation damage may contribute to the development of acquired lymphangioma (8). Given the rarity of retroperitoneal lymphangioma, we reviewed and summarized the clinical, imaging, and pathological characteristics of cases reported over the past decade (**Table 1**).

**Table 1:** Summary of characteristics of retroperitoneal lymphangioma reported in the literature.

Author	Cases	Clinical manifestations	Radiological manifestations	Pathological manifestations	Treatment	Follow-up
Dunev (20)	1	Nausea, vomiting, abdominal pain	18.5 × 14.2 cm, multilocular cystic mass	CD31(+), D2-40(+)	Laparotomy	NM
Rezaee (21)	1	Chronic abdominal pain	7.9 × 7.9 × 12.6 cm, cystic mass	D2-40(+), CD34(+)	Robotic-assisted laparoscopic	1month
Shayesteh (22)	1	Abdominal pain	13 × 4.5 × 12 cm, mixed composition mass containing both fat and fluid	Anastomotic vascular spaces of varying sizes	Laparotomy	NM
Saadi (23)	5	No symptoms 1 case; abdominal pain 4 cases	Unilocular cystic lesions:3 cases; multilocular cystic lesions: 2 cases	NM	Laparotomy	Average of 32.6 months
Mabrouk (24)	1	Abdominal distension, constipation	24.5 × 19.5 × 14.5 cm, cystic mass	Lined by a layer of flattened endothelium	Laparotomy	6 months
Su (25)	1	Abdominal pain, abdominal distension,	24.2 × 16.7 × 27.8 cm, cystic mass	Proliferation of fibrous tissues and vascular	Laparotomy	NM
Nabhan (8)	1	Abdominal pain and dyspepsia	Cystic mass	NM	Laparotomy	12 months
Lim (9)	1	Hematochezia	13 × 9.7 × 15.8 cm, cystic mass	CD31(+), ERG(+), D2-40(+)	Laparotomy	NM
Zhu (26)	1	No symptoms	19 × 8 cm, cystic mass	D2-40(+), CD31(+)	Laparotomy	3 months
Lei (27)	1	Abdominal pain, abdominal distension	35.6 × 23.5 × 13.8 cm, cystic mass	SMA(+), ki-67(+1%), D2-40, CD34(-)	Laparotomy	Recurrence after 6 months
Rajput (11)	1	Abdominal pain	13 × 10 × 12 cm, cystic mass	Lymphatic aggregates in the cyst wall	Laparotomy	1 month
Sabir (12)	1	Abdominal distension	14.6 × 15.8 × 16.4 cm, cystic mass	Interstitial lymphocytic proliferation	Laparoscopic	NM
Liu (15)	15	No symptoms 10 cases, low back pain 2 cases, abdominal pain 1 case, nausea 1 case, blood pressure elevation 1 case	Cystic mass 12 cases, cavernous 3 cases	D2-40(+), CD31(+), CD34(+), SMA(+),	Robotic-assisted 1 case, laparoscopic 14 cases	Average of 48 months

NM, not mentioned.

The clinical manifestations of retroperitoneal lymphangioma are diverse. When the tumor is small, patients are often with no obvious clinical manifestation (12/31). As the tumor enlarges, it may compress or invade adjacent organs. This compression can lead to abdominal pain (12/31) and abdominal distension (4/31). Some patients may also present with symptoms such as nausea, elevated blood pressure, hematochezia, and lower back discomfort (9). In this case, the tumor was large (13 × 10.5 × 7.2 cm). Imaging revealed unclear boundaries between the tumor and the surrounding intestinal canal, with blurring of the periintestinal fat space. Histopathology examination revealed lymphocyte infiltration around the cystic cavity. These findings suggest that in addition to compression symptoms, the tumor also caused inflammatory reactions in adjacent intestines, resulting in aggravated abdominal pain. Moreover, as the tumor enlarges, the risks of intestinal volvulus, tumor rupture with hemorrhage, or infection increase significantly. Patients in such conditions typically present with more severe clinical symptoms and may require more urgent surgical intervention. In general, retroperitoneal lymphangioma lacks specific clinical symptoms, and its definitive diagnosis relies on imaging examinations.

We systematically reviewed the literature and found that most retroperitoneal lymphangiomas are cystic. This may be associated with the loose connective tissue present in the retroperitoneal space. On CT imaging, retroperitoneal lymphangiomas typically appear as low-density, unilocular or multilocular cystic lesions with well-defined margins. Calcification is rarely observed. Literature indicate that the presence of sediment at the bottom of the cyst highly suggests lymphangioma (10). The signal intensity of lymphangiomas on MRI is affected by the cystic contents. These contents may include chylous fluid, serous fluid, hemorrhage, or a mixture of these components. Consequently, the lesions often manifest as long T1 and T2 signals. The enhancement scan revealed significant enhancement of the capsule wall and solid portion. MRI provides soft tissue resolution and can accurately demonstrate the local extent of the tumor. It can also demonstrate invasion of adjacent organs, thereby providing an important basis for the formulation of individualized treatment strategies. When the tumor predominantly contains cystic components, it should be differentiated from common retroperitoneal cystic tumors. These include cystic teratoma, serous cystadenoma, and ovarian cyst. However, when the cystic component is reduced, it becomes difficult to identify lymphangioma on imaging, and sarcoma or neurogenic tumors are more likely to be suspected. In this case, the tumor exhibited a cystic-solid appearance. This finding may be related to the presence of mucoprotein and hemorrhage in addition to lymphatic and chylous fluid within the cyst. As the tumor grows, it envelops retroperitoneal tissues, resulting in the lesion containing components such as fat and smooth muscle. Furthermore, prolonged infection of the tumor leads to thickening and fibrosis of the tumor margins and internal septa. These changes may contribute to an increase in solid components within the tumor. Notably, we observed traversing blood vessels within the tumor. Perivascular inflammatory reactions and increased vascular permeability may lead to dilation of the ileocolic vein with formation of small vascular aneurysms. Although small blood vessels traverse the mass, they do not contribute to the tumor’s blood supply. The degree of tumor enhancement is unrelated to these traversing vessels. This occurs because the lymphangioma enveloped the vessels during its growth process, without invading them. This could be used as a typical radiological sign of this tumor.

Histopathology is the gold standard for diagnosing retroperitoneal lymphangioma. Based on histological differences, lymphangiomas can be classified into capillary lymphangioma, cavernous lymphangioma, and cystic lymphangioma (11). Cystic lymphangioma is the most common type found in the abdominal cavity and retroperitoneal space. Histopathologically, lymphangiomas typically present as dilated thin-walled lymphatic channels lined by D2–40 and Prox1-positive lymphatic endothelial cells. The specific marker for lymphangiomas is D2–40 positive expression, while some lymphangiomas demonstrate CD31 and CD34 expression (12). In this case, morphological examination revealed a multilocular cystic structure lined by flattened endothelial cells and filled with lymphatic fluid. The cyst wall contains smooth muscle fibers. Immunohistochemical staining exhibited positive expression of D2-40, CD34, vimentin, and SMA, confirming the diagnosis of a retroperitoneal cavernous lymphangioma.

Spontaneous regression of lymphangiomas is extremely rare. However, cases have been reported in which the multiple colorectal lymphangiomas regressed spontaneously (13). For small and asymptomatic lymphangiomas, treatment may not be necessary, as increased lymphatic pressure can lead to recanalization of previously obstructed lymphatic vessels. Moreover, increased pressure within the lymphatic vessels may stimulate macrophage proliferation. The phagocytic activity of macrophages has also been proposed as a mechanism underlying the spontaneous regression of lymphangiomas (14). Surgery remains the preferred treatment option for lymphangioma. Surgery allows direct visualization of tumor invasion into adjacent tissues, blood vessels, and nerves, thereby facilitating complete tumor removal. When cystic lymphangioma is large and difficult to resect surgically, puncture and drainage of the cyst can be performed to reduce its volume and facilitate surgical resection. This may decrease surgical difficulty and reduce operative risk. However, complete evacuation of the cystic fluid should be avoided. Maintaining partial tension within the cyst wall can help ensure complete tumor resection (15). When the tumor infiltrates surrounding tissues, it should be resected together during surgery. Previous studies have demonstrated that incomplete resection is a significant risk factor for tumor recurrence. Reported recurrence rates range from 10% to 50% (10, 16). The present patient underwent tumor resection in the right lower abdomen, and the current tumor also originated from the same region. However, due to the considerable time elapsed, the details of specific surgical procedure remain unknown. Consequently, it cannot be determined whether the previous surgical history represented a risk factor for tumor recurrence or induced tumorigenesis. During this surgery, we resected the primary tumor along with the involved cecum, terminal ileum, and right hemicolectomy to prevent tumor recurrence. In addition, studies have reported cyst aspiration and sclerosing agent injection therapy as a viable treatment option. This approach is commonly applied in areas with loose connective tissue, such as the head, neck, and axilla (17, 18). Studies have also revealed that this method is more effective for macrocystic lymphangiomas compared to microcystic lymphangiomas (19). However, the efficacy of sclerotherapy remains uncertain. In addition, the procedure is relatively complex and may cause complications, such as infection and necrosis at the injection site. Consequently, it is only used as a supplementary approach in clinical practice when the tumor is unresectable or incompletely resected.

In summary, primary retroperitoneal lymphangiomas lack specific clinical manifestations. In this case, the imaging findings did not present the typical unilocular or multilocular cystic changes with clear boundaries. Instead, the tumor predominantly appeared as a cystic-solid mass. The lesion extended along the retroperitoneal space, squeezing and infiltrating surrounding tissues, and vessels traversing the tumor, but did not participate in the blood supply. Radiological imaging plays a vital role in assessing tumor size and evaluating the extent of tumor removal before surgery. It is also useful for monitoring tumor recurrence after postoperative treatment. However, its diagnosis relies on pathological examination. Due to the high recurrence rate of tumors, long-term postoperative follow-up is necessary.

## Data Availability

The raw data supporting the conclusions of this article will be made available by the authors, without undue reservation.
